# Hydroxychloroquine reverses the prothrombotic state in a mouse model of antiphospholipid syndrome: Role of reduced inflammation and endothelial dysfunction

**DOI:** 10.1371/journal.pone.0212614

**Published:** 2019-03-14

**Authors:** Sébastien Miranda, Paul Billoir, Louise Damian, Pierre Alain Thiebaut, Damien Schapman, Maelle Le Besnerais, Fabienne Jouen, Ludovic Galas, Hervé Levesque, Véronique Le Cam-Duchez, Robinson Joannides, Vincent Richard, Ygal Benhamou

**Affiliations:** 1 Rouen University Hospital, Department of Internal Medicine, Rouen, France; 2 Normandie Univ, UNIROUEN, INSERM U1096 EnVI, Rouen, France; 3 University of Rouen, Institute for Research and Innovation in Biomedicine, Rouen, France; 4 Rouen University Hospital, Department of Vascular Haemostasis, Rouen France; 5 Univ, Inserm, UNIROUEN, PRIMACEN, Cell Imaging Platform of Normandy, Institute for Research and Innovation in Biomedicine (IRIB), Rouen, France; 6 Inserm U1234, Rouen, France; Max Delbruck Centrum fur Molekulare Medizin Berlin Buch, GERMANY

## Abstract

Antiphospholipid antibodies (aPL) promote endothelial dysfunction, inflammation and procoagulant state. We investigated the effect of hydroxychloroquine (HCQ) on prothrombotic state and endothelial function in mice and in human aortic endothelial cells (HAEC). Human aPL were injected to C57BL/6 mice treated or not with HCQ. Vascular endothelial function and eNOS were assessed in isolated mesenteric arteries. Thrombosis was assessed both *in vitro* by measuring thrombin generation time (TGT) and tissue factor (TF) expression and *in vivo* by the measurement of the time to occlusion in carotid and the total thrombosis area in mesenteric arteries. TGT, TF, and VCAM1 expression were evaluated in HAEC. aPL increased VCAM-1 expression and reduced endothelium dependent relaxation to acetylcholine. In parallel, aPL shortened the time to occlusion and extended thrombus area in mice. This was associated with an overexpression of TF and an increased TGT in mice and in HAEC. HCQ reduced clot formation as well as TGT, and improved endothelial-dependent relaxations. Finally, HCQ increased the p-eNOS/eNOS ratio. This study provides new evidence that HCQ improves procoagulant status and vascular function in APS by modulating eNOS, leading to an improvement in the production of NO.

## Introduction

Antiphospholipid syndrome (APS) is an autoimmune disorder defined by recurrent thrombotic events and miscarriages, with positive antiphospholipid antibodies (aPL) [1]. APS may be isolated (primary APS) or associated to an auto immune disease, most often systemic lupus erythematosus (SLE). Pathogenic effects of aPL were first described *in vitro* and are characterized by endothelial dysfunction, defined by pro-coagulant [2,3] pro-inflammatory [4] and pro-adhesive [5,6] phenotypes.

In parallel we [7] and others [8,9] have reported that flow mediated dilatation (FMD) is decreased in patients with primary and secondary forms of APS, confirming the presence of an endothelial dysfunction in humans. Moreover, our group has recently demonstrated that patients with primary arterial APS also displayed structural arterial changes, associated with a pro-oxidative and pro-coagulant state that was correlated with activation of the TLR2 and TLR4 signalling pathways. Indeed, we demonstrated in an experimental model that the administration to mice of aPL obtained from patients with primary APS, caused marked endothelial dysfunction in small resistance arteries. This alteration was characterized by an altered NO bioavailability, secondary to increased oxidative stress and inflammation [10]. Moreover, all these alterations were prevented by infliximab [11], suggesting a direct effect of TNFα in the pathophysiology of APS.

Hydroxychloroquine (HCQ), an antimalarial drug, is commonly used in rheumatic diseases *i*.*e*. SLE, but some evidences for its interest in APS have recently emerged [12]. Indeed, HCQ reversed platelet activation induced by human aPL [13,14] and protected the annexin A5 anticoagulant shield from disruption by APL. One retrospective case series has shown the interest of HCQ in reducing recurrent thrombosis in patients [12]. However, there is a paucity of data supporting the efficacy of HCQ on coagulation parameters. Indeed only one clinical study has recently reported that HCQ reduced soluble TF levels in APS patients, without any change in thromboelastographic test [15]. Therefore, the aim of our preclinical study is to investigate whether HCQ reverses coagulation parameters and modulates vascular endothelial function, and then protects mice against arterial thrombosis.

## Methods

### Patients and Ig purification

Serum samples were pooled from the serum of six patients with primary arterial APS who fulfilled the international classification criteria for this disease (Table 1), and from 2 healthy blood donors. Written informed consent was obtained from each subject. The study was conducted according to the recommendations set forth by the declaration of Helsinki on biomedical research involving human subjects. The study was approved by the local ethics committee (CPP nord ouest), and all participants provided written informed consent. The study was registers online at https://eudract.ema.europa.eu/results-web/ under the unique identifier Eudract 2015-002182-38.

**Table 1:** Antiphospholipid syndrome patients and antiphospholipid antibody (aPL)-binding characteristics.

	Clinical	Anticardiolipin IgG	AntiB2GP1 IgG	LAC
Patient 1	Stroke	110	599	-
Patient 2	Myocardial infarct	90	2753	-
Patient 3	Stroke	105	1056	-
Patient 4	Stroke	0	4020	-
Patient 5	Renal infarction	115	658	-
Patient 6	stroke	36	429	-

Plasma sample were tested according to the updated criteria (1) for lupus-anticoagulant. Serum aCL IgG autoantibodies and anti-β2GPI IgG were determined by chemiluminescence (QUANTA Flash assays INOVA Diagnostic, Inc) according to the manufacturer’s instructions. APL and non-specific IgG samples were obtained by purification of serum samples using protein G-Sepharose affinity chromatography (Nab prot G spin column, Fischer Scientific France) [11]. The samples were positive for anti-β2GPI-IgG and anticardiolipin IgG when concentrations above 1860 CU and 105 CU, respectively, were measured. Lack of endotoxin was confirmed using the Limulus amebocyte lysate assay (Thermofischer).

### Preparation of HCQ

HCQ tablets (Plaquenil, Sanofi-Aventis, France), each containing 200mg of the molecule, were mashed and solubilized with NaCl 0,9% at 2 different concentrations: 1mg/mL or 10mg/mL, and then kept at 4°C until used.

### Murine model of APS and treatments

Male C57BL/6 mice were obtained from Janvier (Le Genest Saint Isle, France). All experiments were performed in accordance with protocols approved by the Institutional Review Boards. (Comité d’Ethique NOrmandie en Matière d’EXpérimentation Animale ENOMEXA n°54).

APS was induced in mice as described previously [7] by a single i.v. injection of a 300μL (4.2mg) aPL. Control groups received either saline (sham) or 300μL of serum without antiphospholipid activity obtained from healthy donors (NSIg). These injections were given through an insulin syringe (29 G, Myjector U-100 INSULIN). The mice were treated by daily tube-fed with HCQ at 12μg/g for 7 days.

On day 7, mice were anesthetized with 3.6mg/kg xylazine (Rompum 2%, Bayer, France) and 90mg/kg ketamine (Imalgène 1000, Merial, France) intraperitoneally and underwent laparotomy.

### Ex-vivo vascular studies

A first-order division segment of the mesenteric artery was carefully dissected free with a dissecting microscope. Artery segments 1.5–2.5 mm long and 150–250 μm in diameter were mounted in a small myograph for isometric tension recording (Danish Myo Technology A/S, Aarhus, Denmark). Care was taken during the dissection procedure to avoid damage to the endothelium. After equilibration, the vessels were stretched to a physiological tension and allowed to equilibrate for 30 minutes, during which the chamber temperature was progressively increased to 37°C. Normalization procedure was performed as previously described. After a 30-minute equilibration period, segments were precontracted by a single concentration of phenylephrine (10^-5^M) and relaxations induced by increasing concentrations of acetylcholine (10^-9^M to 3.10^-5^M; endothelium-dependent relaxation) or the nitric oxide donor sodium nitroprusside (SNP 10^−9^ to 10^-5^M; endothelium-independent relaxation) were assessed. Rings were washed twice and allowed to equilibrate for 20 minutes between each concentration-response curve. The contribution of NO to the relaxations to acetylcholine was also assessed by 30 min preincubation with the NO synthase inhibitor NG-nitro-L-arginine (L-NNA: 10^−4^ M).

To investigate the possible contribution of NADPH oxidase, mesenteric arteries were incubated with apocynin 10^-4^M for 60 min.

### In vivo thrombosis model

#### Carotid artery thrombosis

Mice were anesthetized as described previously, and the right common carotid artery was exposed. A 1×1 mm strip of filter paper (Whatman n°1) saturated with 10% ferric chloride (FeCl3) was applied directly to the outer vessel side. At 3 min, the strip was removed, and the vessel was washed with saline solution. An ultrasonic flow probe (Transonic T410 model) was placed around the carotid artery immediately above the injured site. Blood flow was then recorded for 30 min or 5 min until complete occlusion of the carotid artery, defined by the absence of detectable flow.

#### Mesenteric artery thrombosis

Mice were anesthetized and the mesentery was exposed. An area containing more than 3 mesenteric divisions was selected for the study. A 10 mm–wide strip of Whatman filter paper was soaked in 10% saturated FeCl3 solution and applied for 1 min to the surface of the mesentery on the selected area. For platelet labelling, 100μl Rhodamine 6G 0.05% and 150μL FITC-dextran 500kDa 0.1% (to label the circulating lumen) were administered through a catheter inserted in the jugular vein. Animal were maintained at 37° by a thermo-controlled plate and the mesentery was perfused with a 37° saline solution. The perfused animal positioned onto a temperature-controlled plate was directly transferred onto a motorized stage of a confocal macroscope (TCS LSI, Leica Microsystems, Nanterre, France). To visualize FITC-dextran and Rhodamine 6G, the tissue was illuminated with a 488 nm or a 532 nm wavelength light by means of a laser diode through a confocal laser scanning macroscope equipped with a x2 dry objective (NAmax: 0.224, working distance: 39 mm, diameter: 58 mm, Leica Microsystems). In a sequential mode, the green fluorescence emission was detected from 500 to 530 nm and the orange fluorescence emission from 550 to 600 nm. Images were recorded before the FeCl3 injury and each minute for 30 min. At the end of the experiment a Z stack acquisition was performed to visualize the entire vascular bed in the analysed area. Lumen (FITC labelled) and the sum of thrombosis area were quantified using image J software. Results were expressed as percentage thrombosis surface / total arterial lumen.

#### TF expression

RNA was isolated from mice aorta using TRIZOL reagent (Invitrogen). Real time (RT)-PCR was performed using primers directed toward TF and 18s RNA (Invitrogen). PCR experiments were performed using a Light Cycler-Fast Start DNA Master SYBR Green I kit (Roche). Quantitative PCR was performed in a total reaction volume of 20 μL per capillary for the Light Cycler format. The number of cycles at which the best-fit line through the log-linear portion of each amplification curve intersects the noise band was inversely proportional to the log of copy number. The resulting 18S values were used as standard for presentation of the mRNA data of the different transcripts. Oligonucleotide primers were designed according to the published sequences: 18S gene was amplified with forward GTGGAGCGATTTGTCTGGTT and CGCTGAGCCAGTCAGTGTAG reverse primers of 200 bp each, Tissue factor gene with forward CATGGAGACGGAGACCAACT and TAAAAACTTTGGGGCGTTTG reverse primers of 69 bp each, eNOS GACCCTCACCGCTACAACAT and CACAGGATGAGGTTGTCCT reverse primers of 77 bp each,

#### ELISA assays

On day 7, the inferior vena cava was punctured using a 1 mL syringe previously impregnated with heparin (heparin Choay 25.000 IU / 5 mL), to withdraw an average of 0.7 mL whole blood. Blood was then centrifuged at 825 g at 4°C, and plasma was frozen at -80°C. Levels of adhesion molecules VCAM-1, E-selectin and TNFα were measured in plasma by sVCAM-1/CD106 Quantikine ELISA Kit, E-Selectin/CD62E Quantikine ELISA Kit (R&D system), and TNFα Quantikine ELISA Kit (R&D system). The plates were read by a BIOTEK ELx800 analyzer (BIOTEK, France).

#### Western blot assays

Carotid or mesenteric arteries were homogenized by mechanical disruption in cold Phosphosate Extraction Reagent (Novagen) lysis buffer. The homogenized tissue was separated by sodium dodecyl sulfate-polyacrylamide gel electrophoresis (7% Criterion XT Tris-Acetate Protein Gel, Bio-Rad Laboratories, Hercules, USA). Total Proteins were visualized via a stain-free gel imaging system (Bio-rad Gel Doc EZ Membranes). Proteins were transferred on membranes by using a Transblot Turbo transfer system (Bio-rad). Membranes were incubated with the following primary antibodies: Anti TF (Abcam AB151748), anti-eNOS (monoclonal; Transduction Laboratories), anti–P-eNOS (monoclonal; serine 1177; Serva, Heidelberg, Germany). Membranes were washed again and incubated with a secondary antibody (Jackson Immunoresearch Laboratories, West Grove, USA). Densities of the specific bands were assessed on a ChemiDoc System with Image Lab Software. Results are presented as a ratio vs. 18S or a P-eNOS/eNOS ratio (to estimate eNOS activation).

#### Thrombin generation

Assessment of thrombin generation in mice has been previously described [16]. Briefly, blood was drawn from the inferior vena cava into a syringe containing 3.2% sodium citrate (1/10 volume). Blood was centrifuged at 2250 g for 15 min and the supernatant was then centrifuged at 13000 g for 5 min to obtain platelet-free plasma, which was frozen at -80°C.

Thrombin generation was measured in duplicate by calibrated automated thrombography using a Fluoroscan Ascent fluorometer (Thermoscientific Labsystems, Helsinki, Finland). Thrombin generation curves and endogenous thrombin potential (ETP) were calculated using the Thrombinoscope software (Thrombinoscope BV, Maastricht, The Netherlands). 20μl plasma was mixed with 1pM tissue factor and 4μM phospholipids (low PPP reagent, Diagnostica Stago, Asnières, France). The reaction was started with 20 μL FLUCAkit (FLUCAkit, Diagnostica Stago, Asnières, France) containing calcium chloride and the fluorogenic substrate (Z-Gly-Gly-Arg-AMC). Activated protein C (APC) was used at 25 nM and APC resistance (APCr) was assessed by quantification of the effect of APC on ETP and standardized on normalized APCr (nAPCR) determined in the presence (ETP+APC) or absence of APC (ETP). The normalization was done with a pool of healthy mice plasma (HMP).

#### Cell culture

Immortalized aortic endothelial cells (TeloHAEC; ATCC CRL-4052, USA) were expanded with Endothelial Cell Growth Kit VEGF (ATCC PCS-100-041, USA) supplemented by 10 units/ml penicillin and 10μg/ml streptomycin (P4333, Sigma Aldrich, France) at 37°C in humidified atmosphere with 5% CO2. Adhesive cultures were developed in 75 cm^2^ culture flasks. At 90% confluence, cells were incubated with trypsin for 5 min at 37°C then Dulbecco's Phosphate-Buffered Saline was added to neutralise trypsin. Cells suspensions were centrifuged 5 min at 150 g and pellets were suspended in culture medium. Cells were seeded at least 24 hours and the confluence was 80–90% in each well prior to incubation with aPL or control IgG.

***Treatment*:** To test the effects of aPL on HAEC, cells were resuspended to a density of 3.10^5^ cells/mL in medium and incubated with aPL (100μg/ml) without or with hydroxychloroquine (1 μg/mL), or a as negative control (nonspecific IgG; 100μg/ml) [14] for 4 hours at 37°. To investigate the possible contribution of NADPH oxidase, HAEC were incubated with niflumic acid (NFA) 0.1mM 4 hours prior to aPL.

***Western Blot***: HAEC were lysed in RIPA buffer (Abcam) and a cocktail of proteases inhibitors were added (Complete Ultra tablets EDTA free from Roche). The primary antibodies used were anti MYD88 (Abcam AB2064), Anti TF (Abcam AB151748) and anti VCAM-1 antibodies (Santa cruz-sc 8304).

***Thrombin generation assay***: Cells were cultured in 96-well plates and allowed to adhere at 37°C in humidified atmosphere with 5% CO_2_. At 90% confluence, cells were incubated for 4 hours with different concentrations of aPL, aPL + HCQ or NS-IgG. Culture mediums were removed and 80μl PPP was added to each well. Thrombin generation was initiated with 1 pM or 5 pM TF and 4 μM aPL. Thrombin generation was performed the same day for each condition, in order to reduce the variation of thrombin generation due to variable confluence.

***Flow cytometry***: HAEC were incubated with aPL for 4 hours, after which staining was performed with PE/Cy7 anti-human CD141 (thrombomodulin clone 80 BioLegend). Cohesive cells were incubated with acutase.

#### Data analysis

All results are expressed as mean ± SEM. In all *in vitro* experiments, n represents the number of animals from which the arteries were taken (5 to 8 mice). For quantitative variables, i.e. ETP, peak thrombin, VCAM-1, E-selectin expression, data were analyzed by ANOVA followed when significant by a Tukey post hoc test. Contractile responses are expressed in millinewtons (mN) and normalized to vessel length (mN/mm), and relaxing responses are expressed as percentage of reversal of the contractions induced by phenylephrine. ANOVA for repeated measures was used to determine the concentration–response curves and in the study of the carotid blood flow. Difference between groups was analysed by a Student t-test when appropriate. Data were analyzed using Prism 5.0 from Graph Pad Software. A *P* value ≤ 0.05 was considered statistically significant.

## Results

### aPL–induced prothrombotic state and inflammation is reversed by HCQ

**Fig 1:**
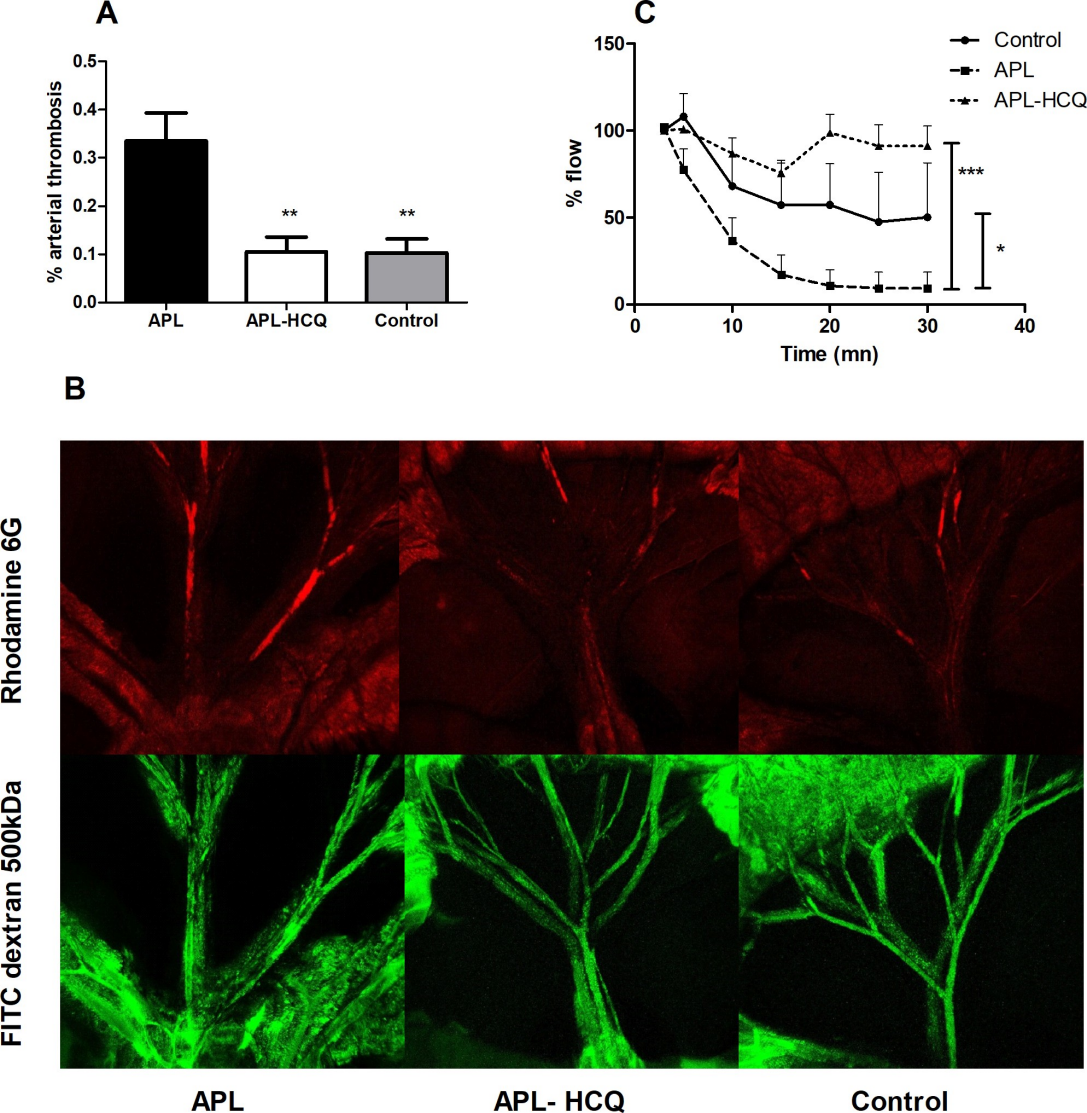
Effects of Hydroxychloroquine (HCQ) on in vivo thrombosis. (A) The thrombosis/arterial area ratio in control mice, or aPL mice either untreated or treated with HCQ. 30min after chemical injury (FeCl3 10%). (B) thrombosis was assessed with macroconfocal imaging. Platelets were labeled with rhodamine6G (red) and total arterial area was labeled with FITC-dextran 500kDa FITC (green) (n = 5). (C) Carotid blood flow (n = 6) measured with a flow probe in mice before and 30 min after FeCl3 injury. Values are mean±SEM *: P<0.05; **: P<0.01 ***: P<0.001.

In mesenteric arteries. aPL mice had a larger clot to arterial surface ratio, compared to control (aPL 33.53±5.78% *vs*. control 10.3±2.90%; P<0.01), (Fig 1A and 1B). HCQ significantly reduced mesenteric thrombosis (aPL-HCQ 10.53±3.08%; *P<0*.*01 vs*. *aPL*).

In the carotid thrombosis model (Fig 1C), carotid flow rapidly decreased more rapidly in the APL group, compared to control (P<0.05), while no occlusion appeared after 30 min of follow-up in the aPL-HCQ group. (P<0.001).

**Fig 2:**
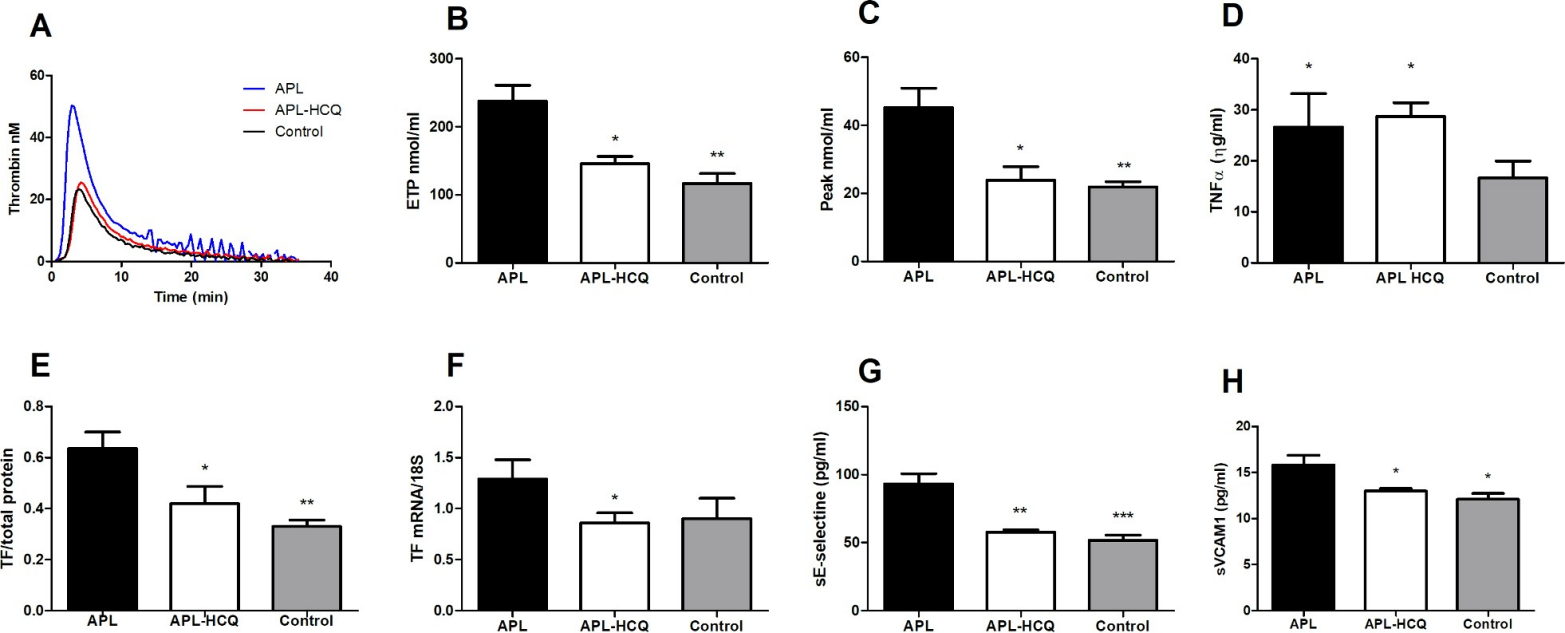
Effects of aPL and HCQ on procoagulant and inflammatory properties in mice. (A) Thrombin generation curves, (B)endogenous thrombin potential, (C) peak thrombin measured by calibrated automated thrombography (CAT) in the presence of Activated C protein (APC).(D) Plasma levels of TNFα, (E) carotid Tissue Factor expression assessed by western blotting and (F) aortic TF mRNA expression assessed by RT-PCR. (G) Plasma levels of sE-selectin, and (H) sVCAM-1 assessed by ELISA. Values are mean±SEM *: *P<0*.*05*; **: *P<0*.*01* ***: *P<0*.*001;* n = 5 / group.

**Fig 3:**
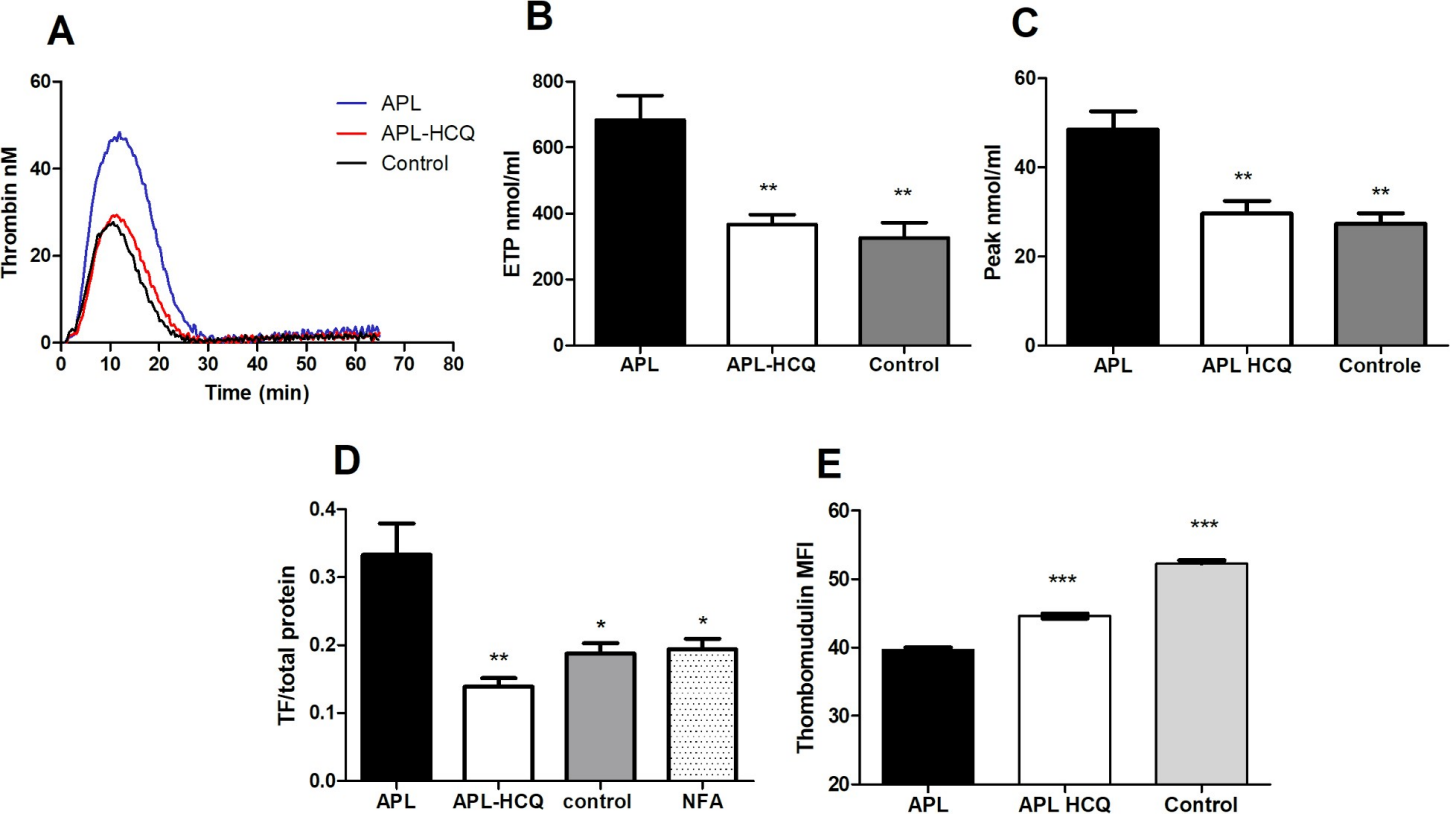
Effects of HCQ on procoagulant and inflammatory properties in endothelial cells. (A)Thrombin generation curves, (B) endogenous thrombin potential, and (C) peak thrombin measured in the presence of Activated C protein (APC) by calibrated automated thrombography (CAT) in HAEC. (D) Tissue factor expression measured in HAEC with or without niflumic acid 0.1mM by western blotting (E)Thrombomodulin quantified by flow cytometry assay, expressed in mean fluorescence intensity. (MFI) Values are mean±SEM *: *P<0*.*05*; **: *P<0*.*01* ***: *P<0*.*001;* n = 5 / group.

Western blot analysis showed that untreated aPL mice displayed a marked increase in TF levels compared to controls (aPL 0.63±0.07; control 0.32±0.03; *P<0*.*01*) and this was reduced by HCQ (aPL-HCQ 0.42±0.26; *P<0*.*05 vs*. *aPL*) (Fig 2E). Furthermore, when compared to control, TF mRNA expression tended to increase in aPL mice (aPL 1.29±0.19; control 0.90±0.19; *P = 0*.*21*) while HCQ reduced this expression (0.86±0.09; P = 0.048 *vs*. *aPL)* (Fig 2F). Finally, TF expression increased in HAECs after 2h exposure to aPL (supplemental data), and this was significant avec 4h (aPL 0.33±0.04; control 0.18±0.04; *P<0*.*05*). HCQ significantly decreased TF expression, that returned to baseline values (aPL-HCQ 0.14±0.01; *P<0*.*01 vs*. *aPL)* (Fig 3D). The endosomal NADPH oxidase inhibitor NFA (0.1mM), reduced TF expression that returned to baseline values suggesting that the polyclonal antibodies used were activating the NADPH oxidase pathway (aPL-NFA 0.19±0.02 *P<0*.*05 vs*. *aPL)*,(Fig 3D).

Results with assessment of thrombin generation in mice are shown in Fig 2. aPL mice displayed a significant increase in ETP (aPL: 237.40±23.78; control: 116.3±14.81 *P<0*.*01*) and peak thrombin generation (aPL 45.23±5.67; control 21.88±1.57 *P<0*.*01*). HCQ induced a significant decrease in ETP (146±11 *P<0*.*05 vs*. *aPL*) and peak (23.89±3.10 *P<0*.*05 vs*. *aPL*) suggesting a reduced procoagulant state.

Results on thrombin generation test in endothelial cells HAEC are shown in Fig 3. Compared to control, aPL significantly increased ETP (aPL 683.40±74.11; control 326.50±45.82; *P<0*.*01*)and peak thrombin (aPL 48.52±4.11; control 27.37±2.36; *P<0*.*01*). HCQ markedly decreased both ETP (aPL-HCQ 367.00±29.45; *P<0*.*01 vs*. *aPL*) and peak thrombin generation (aPL-HCQ 29.61±2.86; *P<0*.*01 vs*. *aPL*

Finally, as we used a high concentration of TF to trigger thrombin generation, we investigated the surface expression of thrombomodulin on HAEC, in order to explain the excess of ETP (Fig 3E). aPL significantly decreased TM expression (aPL 39.68±0.35; control 52.30±0.40; *P<0*.*001*), and this was reversed by HCQ significantly improved the TM expression (aPL-HCQ 44.63±0.37; P<0.001 vs aPL.).

### aPL–induced endothelial dysfunction is reversed by HCQ

**Fig 4:**
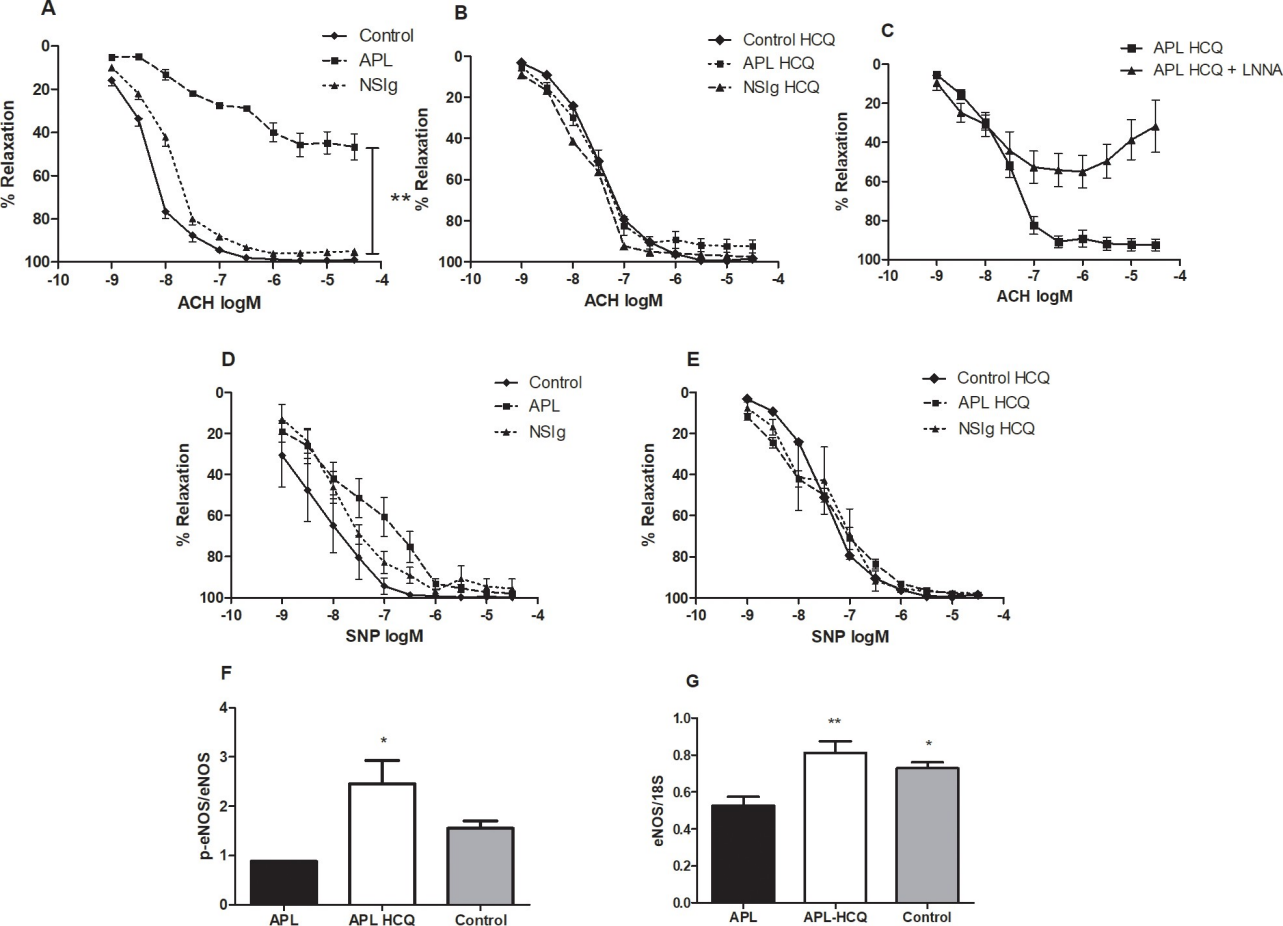
Effect of antiphospholipid antibodies and hydroxychloroquine on the relaxing responses to acetylcholine and on eNOS. (A,D) Endothelial function of mesenteric arteries was assessed seven days after the injection in control animals (full lines) and in aPL-IgG mice either untreated (dotted lines) or treated with HCQ (dashed lines) Relaxation responses to ACH and SNP in untreated mice or (B,E) HCQ-treated mice. (C) Effect of LNNA in the relaxing response to ACH, (F) Mouse mesenteric artery ratio of P-eNOS/eNOS measured by western blot. (G) Mouse aortic eNOS mRNA expression. Values are mean±SEM *: *P<0*.*05*; **: *P<0*.*01* ***: *P<0*.*001;* n = 8 by groups in relaxation and ELISA experiments, n = 4 in western blot and qRT-PCR assays.

We first studied endothelial function by measuring the relaxation induced by increasing concentrations of acetylcholine (10–9 to 3.10^-5^M) in mouse mesenteric arteries (Fig 4A). In untreated mice, aPL reduced the responses to acetylcholine compared to control and NSIg (control 99±1%; NSIg 97±0.2%; APS 47±6%; *P<0*.*001*) while responses to SNP were similar in all groups (Fig 4D). This demonstrates that aPL induces severe endothelial dysfunction in untreated mice.

In aPL mice, HCQ induced a significant improvement in the relaxing response to acetylcholine, as compared to untreated mice (aPL-HCQ 96±1%; *P<0*.*001 vs*. *aPL*). Indeed, the relaxations to acetylcholine in HCQ-treated aPL mice were not significantly different from those of control and NSIg (Fig 4B). Furthermore, HCQ did not affect the response to ACH and SNP in control mice (Fig 4B and 4E). The relaxing response in treated mice was markedly reduced by the NO synthase (NOS) inhibitor L-NNA (10^−4^ M) demonstrating that the improvement in relaxation induced by HCQ is largely due to an increased NO-mediated relaxation (Fig 4C). Previous studies have demonstrated that aPL-induced endothelial dysfunction was mediated by inhibition of eNOS activation [17] and mRNA expression[11]. To determine whether hydroxychloroquine normalize eNOS activation we measured the P-eNOS/eNOS ratio (Fig 4F) and eNOS mRNA expression (Fig 4G) in mesenteric arteries and aortas in mice. HCQ induced a significant increase in P-eNOS /eNOS ratio (aPL 0.87±0.08; aPL-HCQ 2.46±0.47 P = 0.038) and in eNOS mRNA expression (aPL 0.53±0.05; aPL-HCQ 0.73±0.03).

In addition to the assessment of the vascular function, we measured the effect of HCQ on inflammatory biomarkers. Compared to control, APL mice displayed a marked increase in soluble levels of VCAM-1 (control 12.08±0.64, aPL 15.81± 1.04; *P<0*.*01*) and E-selectin (control 51.58±4.00; aPL 93.05± 7.58; *P<0*.*001*; Fig 2G and 2H). In contrast, when compared to untreated mice, HCQ induced a significant drop in levels of sVCAM-1 (aPL-HCQ 12.99±0.29; *P<0*.*05 vs*. *aPL)* and sE-selectin (APL-HCQ 57.70±1.81; *P<0*.*01 vs*. *aPL*). Finally, aPL increased plasma TNFα (aPL26.60±6.54; Control 16.65±3.26; P<0.01), however this was not affected by HCQ (aPL-HCQ 28.75±2.65).

## Discussion

The present study demonstrates the beneficial effects of HCQ on thrombotic status in APS, evidenced by reduced clot formation and thrombin generation in both a mouse model and in human endothelial cells. One most likely mechanism for these beneficial effects is the improvement of endothelial function (restored NO production), associated with correction of the pro-inflammatory phenotype observed both *in* vivo and in vitro.

Although APS is primarily characterized by thrombosis and obstetric morbidities [1], its prothrombotic status has rarely been addressed. Several studies reported increased monocyte TF expression in APS patients[18–20]. A few in vitro experiments also support a key role for aPL in the induction of TF expression [21–24], as confirmed in the present study. The expression of TF by HAEC increased after 2 hours and was significant after 4 hours incubation with aPL. This incubation time has previously been reported by Pierangeli et *al* in HUVEC [25]. More recently, Muller-Calleja et al. found that the time course of TNFα release by monocyte was dependent on the type of aPL. Indeed, TNFα release by MonoMac cells in the presence of HL5B and J7G aPL was maximum between 3 and 6 hours. Our results are also consistent with those of Prinz et al. showing that HL5b antibodies induced endothelial TF mRNA expression starting at 2 hours and peaking at 4 hours [26]. Since TF triggers the extrinsic pathway of the coagulation cascade leading to the production of thrombin, we then assessed aPL-induced thrombin generation. Therefore, the significant increase in ETP and peak thrombin generation in mice is consistent with the changes in TF and appears more pathologically relevant, since the thrombin generation test evaluates the overall activation and inhibition of haemostasis [27]. Indeed, some reports suggest that detection of an altered thrombin generation under in vitro conditions might closely mimic what occurs in vivo and then provide a good estimation of the total coagulation potential, compared to traditional coagulation tests [28]. Our results are in line with clinical studies describing an association between an unbalanced thrombin generation and thrombotic risk in APS [29–31].

In addition, since a recent study suggests that the prothrombotic effect of anti-β2GP1 antibodies involves both the expression of TF and TM in endothelial cells [32], we assessed thrombomodulin expression in HAEC to determine whether aPL increase ETP and peak thrombin generation test despite a high concentration of TF in our experiments. Thus, the decreased expression of TM after exposure to aPL that is partially reversed by HCQ confirms its important role in the thrombin generation in APS and suggests a potential mechanism by which HCQ prevents thrombosis. This is confirmed by the results in the *in vivo* models of thrombosis, where HCQ reversed FeCl_3_-mediated thrombosis. Therefore, we demonstrated that HCQ improves the TF expression, thrombin generation and *in vivo* thrombosis. Edwards *et al*. previously found that the thrombotic profile was improved in APS mice treated with HCQ [33]. Recently, the results of an open-label study confirmed in patients receiving HCQ for 3 months a reduction in soluble TF levels, suggesting an antithrombotic effect. However, they failed to show any effect on thromboelastography [15]. Therefore, to date the effects of HCQ on the thrombin generation test have not been reported in clinical studies. Indeed, Cohen *et al*. in the RAPS study, were unsuccessful to use thrombin generation as primary endpoint to investigate the efficacy of an antithrombotic treatment [34].

Because endothelial dysfunction is a well-recognized contributor of the procoagulant status in APS, we investigated the endothelial effect of HCQ [7]. This dysfunction is directly mediated by aPL, as demonstrated by transfer experiments in cell cultures [25] and in mice [11]. Herein, our experiments confirm that aPL induce an endothelial dysfunction as demonstrated both by the overexpression of adhesion molecule VCAM and E selectin and the altered endothelium dependent relaxation to acetylcholine. The results of the inhibitory effect on vascular relaxation by L-NNA confirmed the central role of NO in the aPL–induced endothelial dysfunction and its improvement by HCQ. This hypothesis is also supported by the observed decrease in an index of NOS activation (mesenteric phosphorylated S1177 eNOS/eNOS ratio) in APL mice that is reversed by HCQ. Recently preclinical studies indicated that APS-related thrombosis was due to eNOS inhibition via apolipoprotein E receptor 2 (apoER2)-dependent processes. Some reports demonstrated that, in endothelial cells and in mice, aPL activates the protein phosphatase 2A (PP2A) leading to a dephosphorylation of S1179 (S1179 in bovine cells, S1177 in human) and finally to decreased NO production [17,35].

Recently, a study demonstrated that HCQ may interfere with the TNFα pathway in endothelial cells directly exposed to this cytokine [36]. We previously reported that vascular dysfunction in APS was largely mediated by TNFα since treatment with infliximab improved endothelial function through modulation of the NO pathway. We can assume that the effect of HCQ is partially secondary to its anti TNFα properties. However, we previously failed to demonstrate an effect of infliximab on eNOS mRNA levels [11]. In this study, HCQ significantly improved eNOS mRNA expression and protein content, suggesting that it may stimulate transcription or posttranscriptional regulation of eNOS expression.

Our results should be analyzed in regard to the recent publication of Müller et al. who showed that the signaling pathway of aPL was epitope-dependent. It is interesting to note that HCQ affected only the anticardiolipin antibody (HL5b) which is cofactor-independent as well as the one that recognizes both cardiolipin and B2GP1 (J7G).

The possible role of NADPH oxidase inhibition in the antithrombotic effects of HCQ has been demonstrated previously [37]. In our study, we have also found that NFA, an endosomal NADPH inhibitor, prevented aPL-induced TF overexpression in HAEC [38], suggesting that NADPH oxidase is the cornerstone of endothelial dysfunction mediated by aPL, and of the protective effect of HCQ.

One potential limit of our study is that it used used a preparation containing different subpopulations of aPL however, the used of pathogenic aPL may closely represent the clinical setting, where most APS patients have more than one aPL species.

## Conclusion

The present study demonstrates the beneficial effect of HCQ on the prothrombotic state, most likely related to the improvement of endothelial function. Potential mechanisms include improvement of thrombomodulin expression and restored eNOS activation via inhibition of endosomal NADPH oxidase. Therefore, HCQ may be of special relevance in clinical practice and, this hypothesis warrants evaluation. In this regard, we are currently conducting a randomized controlled trial (APLAQUINE) that investigates whether treatment with HCQ modulates vascular endothelial function and coagulation parameters in patients with APS (*NCT02595346*).
